# "Acute Kidney Injury predictive models: advanced yet far from application in resource-constrained settings."

**DOI:** 10.12688/f1000research.122344.1

**Published:** 2022-06-13

**Authors:** Busisiwe Mrara, Fathima Paruk, Olanrewaju Oladimeji

**Affiliations:** 1 Anaesthesiology and Critical Care, Walter Sisulu University, Mthatha, Eastern Cape, 5099, South Africa; 2 Critical Care, University of Pretoria, Pretoria, Gauteng, 0001, South Africa; 3 Public Health, Walter Sisulu University, Mthatha, Eastern Cape, 5099, South Africa

**Keywords:** Acute Kidney Injury, predictive models, resource-constrained settings

## Abstract

Acute kidney injury (AKI) remains a major cause of morbidity and mortality in hospitalized patients, particularly critically ill patients. It poses a public health challenge in resource-constrained settings due to high administrative costs. AKI is commonly misdiagnosed due to its painless onset and late disruption of serum creatinine, which is the gold standard biomarker for AKI diagnosis. There is increasing research into the use of early biomarkers and the development of predictive models for early AKI diagnosis using clinical, laboratory, and imaging data. This field note provides insight into the challenges of using available AKI prediction models in resource-constrained environments, as well as perspectives that practitioners in these settings may find useful

## Background

Acute kidney injury (AKI) epidemiology in low-resource settings is underreported due to difficulties with paper-based reporting and diagnosis confirmation due to limited access to laboratory testing. This has been identified as one of the barriers to the advancement of global initiatives aimed at eliminating preventable AKI deaths by 2025. ^1^ Furthermore, epidemiologic research on the development of predictive models of AKI in resource-limited settings is lacking; the few publications on the subject are validations of models developed in well-resourced countries.

Several predictive models for the early prediction of AKI in critically ill patients have been developed, utilizing patient data available in intensive care units (ICUs) and, more recently, machine-learning algorithms. ^2^^,^^3^ The silent and delayed onset of AKI makes early intervention and management difficult, resulting in the progression to dialysis-requiring renal impairment and chronic kidney disease, which is an unaffordable cost in resource-constrained healthcare systems. It is hoped that early detection will allow for interventions such as reducing the impact of nephrotoxic drugs and fluid titration.

Most AKI prediction models have been developed with predictors based on susceptibilities like chronic comorbidities and exposures such as surgical procedures and sepsis. These models had variable performance in the early prediction of AKI; however, their combination with biomarkers improved their predictive performance and focused biomarker use on patients with a high pre-test probability of AKI, thus streamlining biomarker use in the determination of AKI risk. ^4^^,^^5^

## Challenges with models’ application in resource-constrained settings

Despite these advances, some models have been criticized for methodological flaws such as using creatinine as both a predictor and an outcome, having low rates of AKI in the development cohort, using single-centre data, and lacking validation. ^6^ Furthermore, there is limited data on the models’ use for the intended purpose of directing interventions to prevent further kidney injury, presumably due to difficulties with the models’ multiple variables. The models predict AKI up to 24 hours ahead of time, a short timeframe that may allow for changes in medication and fluid prescription but is unlikely to have a significant impact on an already evolving injury process.

The published models integrated into health information systems with electronic alerts have not consistently demonstrated appreciable effects on AKI outcomes. ^3^ Electronic health records are prohibitively expensive in resource-constrained settings. The application of AKI bundle interventions has yielded mixed results in terms of benefit in reducing AKI rates, ^7^^,^^8^ with even less evidence of benefit from individual interventions such as avoidance of nephrotoxins and overzealous fluid resuscitation, raising the possibility of heightened awareness and improved care quality as the reason for improvement rather than the interdependence of the interventions.

For various concerns, the applicability of currently available predictive models in low-resource contexts remains debatable and needs to be refined. Patients in low-resource settings are frequently sicker (due to delayed presentation, limited access to health care or ICU, or both), younger, and have comorbid communicable diseases. Advanced HIV-related illnesses are common, which may influence the occurrence and complications of AKI. HIV is a significant AKI predictor that should be investigated in AKI predictive models for developing countries. The prevalence of HIV in South Africa is as high as 21% in some areas, ^9^ compared to 5% in the USA, where some of the AKI risk models were developed.

Additionally, HIV illness is comparatively more severe due to late presentation and regulated antiretroviral treatment initiation. Hence, research into HIV as a risk factor and its impact on AKI development in patients with severe acute illness requiring ICU admission is critical. Several researchers have identified HIV infection as an independent risk factor for AKI ^10^; the risk is associated with HIV progression as measured by CD4 count and viral load, tenofovir disoproxil fumarate treatment, and hepatitis C co-infection. Other risk factors include the use of herbal and traditional medications with unknown nephrotoxic potential, as well as the high prevalence of infectious disease, traumatic injuries, and pregnancy-related hypertensive disorders. The disparities in AKI epidemiology and causation between high and low-income settings may also be influenced by health-care quality, which is linked to healthcare funding. As a result, the participants and predictors used to develop AKI prediction models in high-income settings are theoretically distinct from those prevalent in resource-constrained settings. 

Furthermore, because the impact and practicability of these predictive tools in high-income settings has not been thoroughly studied, models that are simple to use and incorporate concrete actions to prevent AKI would be advantageous. The cost of the biomarkers, including importation and implementation with specialized laboratory equipment and expertise, is also a barrier to implementation in resource-constrained settings where basic laboratory tests such as 24-hour serum creatinine are difficult to achieve.

## Conclusion

While AKI predictive modelling in high-income health systems is rapidly evolving, lower-income health systems should carefully consider the applicability and costs of these models in resource-constrained settings, unless the resources are abundant. Otherwise, most resource-constrained settings should concentrate on raising awareness about AKI risk, meticulous patient monitoring, careful drug and fluid prescription practice, and general measures to improve health care quality, which is all that is currently feasible.

## Author contributions

BM and OO initiated discussion of the idea; BM and OO created the first draft. BM, OO, and FP critically reviewed and approved this final version.

## Data availability

### Underlying data

No data are associated with this article.
