# Supplementary material for: "Acute Kidney Injury predictive models: advanced yet far from application in resource-constrained settings."
Source: F1000Res. 2022 Jul 13;11:642. Originally published 2022 Jun 13. [Version 2] doi: 10.12688/f1000research.122344.2 (PMC9301258; doi:10.12688/f1000research.122344.2)
Supplement: Peer review history

# Peer review: Reviewer response for version 2

**DOI:** 10.5256/f1000research.136033.r144207

**Published:** 2022-07-20

**Authors:** Nolubabalo Unati Nqebelele

**Affiliations:** 1 Department of Internal Medicine, Faculty of Health Sciences, University of the Witwatersrand, Johannesburg, South Africa

All comments have been adequately addressed by the authors.

Is the topic of the opinion article discussed accurately in the context of the current literature?

Yes

Are arguments sufficiently supported by evidence from the published literature?

Partly

Are all factual statements correct and adequately supported by citations?

Partly

Are the conclusions drawn balanced and justified on the basis of the presented arguments?

Yes

Reviewer Expertise:

Nephrology, Chronic kidney disease, Acute Kidney disease

I confirm that I have read this submission and believe that I have an appropriate level of expertise to confirm that it is of an acceptable scientific standard.

# Peer review: Reviewer response for version 2

**DOI:** 10.5256/f1000research.136033.r144208

**Published:** 2022-07-20

**Authors:** Jeffrey Lipman

**Affiliations:** 1 Jamieson Trauma Institute, Royal Brisbane and Women's Hospital, Herston, QLD, Australia

In my opinion, the manuscript in its current revision is acceptable for indexing.

Is the topic of the opinion article discussed accurately in the context of the current literature?

Yes

Are arguments sufficiently supported by evidence from the published literature?

Partly

Are all factual statements correct and adequately supported by citations?

Partly

Are the conclusions drawn balanced and justified on the basis of the presented arguments?

Partly

Reviewer Expertise:

Intensive Care

I confirm that I have read this submission and believe that I have an appropriate level of expertise to confirm that it is of an acceptable scientific standard.

# Peer review: Reviewer response for version 1

**DOI:** 10.5256/f1000research.134322.r140611

**Published:** 2022-06-30

**Authors:** Nolubabalo Unati Nqebelele

**Affiliations:** 1 Department of Internal Medicine, Faculty of Health Sciences, University of the Witwatersrand, Johannesburg, South Africa

This is a clearly articulated opinion article on the challenges encountered with the use of AKI predictive models in resource-constrained settings. These models have gained popularity and are rapidly evolving in well-resourced environments. The authors discuss the challenges with the general applicability of these models in low-resourced settings. 

 In paragraph 2, the authors discuss the 'unaffordable cost of dialysis-requiring renal impairment in resource-constrained healthcare systems', without providing a reference. There are various publications on this issue and a reference should be provided.

 In the challenges with the models' application in resource-constrained settings (paragraphs 6-7), there is a strong emphasis on HIV infection. Though important and should perhaps be considered in AKI predictive models, the prevalence of HIV varies extensively, even in low-resourced settings and this should be considered in the discussion.

 It is commendable that the authors conclude that raising awareness of AKI and paying attention to patient monitoring and care is where most attention should be placed in resource-limited settings at this stage. Furthermore, the availability of less-than-ideal creatinine should be improved upon in resource-constrained healthcare systems.

Is the topic of the opinion article discussed accurately in the context of the current literature?

Yes

Are arguments sufficiently supported by evidence from the published literature?

Partly

Are all factual statements correct and adequately supported by citations?

Partly

Are the conclusions drawn balanced and justified on the basis of the presented arguments?

Yes

Reviewer Expertise:

Nephrology, Chronic kidney disease, Acute Kidney disease

I confirm that I have read this submission and believe that I have an appropriate level of expertise to confirm that it is of an acceptable scientific standard, however I have significant reservations, as outlined above.

# Response

**Published:** 2022-07-09

**Authors:** busisiwe Mrara

**Affiliations:** Walter Sisulu University, South Africa

Thank you for your valuable input.

 We have added a reference supporting the assertion regarding the astronomical costs of dialysis.

 We accept that HIV prevalence is variable in low-income settings. We have reworded the paragraph to delink HIV prevalence and low-income settings. However, we would like to emphasise that the advanced presentation of HIV and its complications including Acute Kidney Injury, could be an issue in areas of inadequate health care access.

# Peer review: Reviewer response for version 1

**DOI:** 10.5256/f1000research.134322.r140612

**Published:** 2022-06-22

**Authors:** Jeffrey Lipman

**Affiliations:** 1 Jamieson Trauma Institute, Royal Brisbane and Women's Hospital, Herston, QLD, Australia

This is a nice description of the problems of Acute Kidney Injury (AKI) prediction models. As the authors state, AKI is not an uncommon phenomenon in ICU and potentially its prediction could be used to mitigate the consequences of AKI.

 Creatinine is the common biomarker used for kidney function and hence used in the diagnosis of AKI. Its problems in kidney injury are commonly known, a crude marker with a delayed response – today’s creatinine is yesterday’s renal function. In this opinion piece, the authors quite correctly point out the problems of using creatinine in the prediction of AKI, both from the point of view of its delayed response and that it is used in the prediction and the outcome of AKI.

 I have merely one comment - the authors are from South Africa which could be described as a low-income/low-resource setting/region (accepted). In South Africa, there is a large HIV "burden". This may not be so in all similar low-resource regions. The authors link low resources with HIV infections – I would suggest this not to be the case and the authors should “delink” the two issues. Sure bring up the problems of HIV and kidney involvement, but separate the two issues for this manuscript.

Is the topic of the opinion article discussed accurately in the context of the current literature?

Yes

Are arguments sufficiently supported by evidence from the published literature?

Partly

Are all factual statements correct and adequately supported by citations?

Partly

Are the conclusions drawn balanced and justified on the basis of the presented arguments?

Partly

Reviewer Expertise:

Intensive Care

I confirm that I have read this submission and believe that I have an appropriate level of expertise to confirm that it is of an acceptable scientific standard, however I have significant reservations, as outlined above.

# Response

**Published:** 2022-07-09

**Authors:** busisiwe Mrara

**Affiliations:** Walter Sisulu University, South Africa

Thank you for sharing your insights.

 It is correct that the prevalence of HIV is not directly linked to low-resource environments, and is not high in some low and middle-income countries. The problem we would like to emphasise is the advanced presentation of HIV in settings where health care access is inadequate and the consequent possible impact on the occurrence of Acute Kidney Injury.  
